# A joint ventricle and WMH segmentation from MRI for evaluation of healthy and pathological changes in the aging brain

**DOI:** 10.1371/journal.pone.0274212

**Published:** 2022-09-06

**Authors:** Hans E. Atlason, Askell Love, Vidar Robertsson, Ari M. Blitz, Sigurdur Sigurdsson, Vilmundur Gudnason, Lotta M. Ellingsen

**Affiliations:** 1 Dept. of Electrical and Computer Engineering, University of Iceland, Reykjavik, Iceland; 2 Dept. of Medicine, University of Iceland, Reykjavik, Iceland; 3 Dept. of Radiology, Landspitali—University Hospital, Reykjavik, Iceland; 4 Dept. of Radiology, University Hospitals, Case Western Reserve University, Cleveland, OH, United States of America; 5 The Icelandic Heart Association, Kopavogur, Iceland; 6 Dept. of Electrical and Computer Engineering, The Johns Hopkins University, Baltimore, MD, United States of America; Radboud University Nijmegen: Radboud Universiteit, NETHERLANDS

## Abstract

Age-related changes in brain structure include atrophy of the brain parenchyma and white matter changes of presumed vascular origin. Enlargement of the ventricles may occur due to atrophy or impaired cerebrospinal fluid (CSF) circulation. The co-occurrence of these changes in neurodegenerative diseases and in aging brains often requires investigators to take both into account when studying the brain, however, automated segmentation of enlarged ventricles and white matter hyperintensities (WMHs) can be a challenging task. Here, we present a hybrid multi-atlas segmentation and convolutional autoencoder approach for joint ventricle parcellation and WMH segmentation from magnetic resonance images (MRIs). Our fully automated approach uses a convolutional autoencoder to generate a standardized image of grey matter, white matter, CSF, and WMHs, which, in conjunction with labels generated by a multi-atlas segmentation approach, is then fed into a convolutional neural network to parcellate the ventricular system. Hence, our approach does not depend on manually delineated training data for new data sets. The segmentation pipeline was validated on both healthy elderly subjects and subjects with normal pressure hydrocephalus using ground truth manual labels and compared with state-of-the-art segmentation methods. We then applied the method to a cohort of 2401 elderly brains to investigate associations of ventricle volume and WMH load with various demographics and clinical biomarkers, using a multiple regression model. Our results indicate that the ventricle volume and WMH load are both highly variable in a cohort of elderly subjects and there is an independent association between the two, which highlights the importance of taking both the possibility of enlarged ventricles and WMHs into account when studying the aging brain.

## Introduction

As we age, the brain undergoes progressive brain atrophy and the risk of neurodegenerative diseases and cognitive decline increases [1]. Alzheimer’s disease and cerebrovascular diseases [2] are two of the most common causes of dementia, although there are many other causes [3]. Many of these diseases cause changes in the brain that may be visible long before onset of dementia [4]. Neurodegenerative diseases can cause region specific atrophy and lesions that are visible in structural magnetic resonance images (MRIs) [5]. Changes associated with vascular dementia include white matter hyperintensities (WMHs) of presumed vascular origin, lacunar infarcts, and enlarged perivascular spaces [6]. Enlargement of the ventricles may occur due to atrophy or impaired cerebrospinal fluid (CSF) circulation [7]. Both WMHs and enlarged ventricles are also biomarkers for other conditions, e.g., genetic diseases [8] and autoimmune diseases, such as multiple sclerosis [9]. Hence, neurodegenerative diseases and normal aging can cause both WMHs and enlarged ventricles. Early detection of neurodegenerative diseases by use of neuroimaging biomarkers, such as WMH load or ventricle volume, is important to aid in understanding the pathogenesis of these diseases, and make strides towards therapeutics development. Robust detection at early stages enables investigators to start testing possible therapeutic strategies and select presymptomatic patients for clinical trials [10].

To investigate causes of dementia using brain MRI, various structural biomarkers must be analysed, including volumes, shapes, and location in the brain. Biomarkers should not be looked at in isolation when there may be other causes of similar cognitive or physical impairment that present with different biomarkers in the same subject [11]. Furthermore, it may be difficult to distinguish abnormal size of structures, such as the ventricles, because the size may also depend on factors that are not caused by disease, such as age, sex, and intracranial volume [12]. Information from large data sets of brain MRIs can help elucidate biomarkers that better predict abnormality.

The use of robust, accurate, and automated brain segmentation methods is crucial when using specific brain structures as biomarkers, especially at early stages of the disease, when structural changes may be very subtle and hard to identify visually from MRI. Conventional whole brain segmentation methods include atlas based methods [13–16], such as multi-atlas segmentation methods that use deformable registration of multiple annotated atlas images to the subject at hand. A key challenge when using the multi-atlas segmentation approaches is that the size and location of WMHs varies greatly between subjects and hence, they cannot be accurately registered from one subject to another [17–19]. Also, multi-atlas segmentation methods often rely solely on T1-weighted (T1-w) images, which do not provide as good WMH lesion contrast as Fluid-Attenuated Inversion Recovery (FLAIR) images. Finally, multi-atlas segmentation methods can fail when presented with severely enlarged ventricles [20]. Automatic labelling of WMHs and the ventricles can also be challenging using multi-contrast methods due to pulsation artifacts i.e., hyperintense regions resembling lesions within the ventricles, which can appear in FLAIR images. Increasing ventricle size and age have been associated with the severity of these artifacts [21].

The majority of current state-of-the-art brain segmentation methods are based on convolutional neural networks (CNNs) [22–28]. The U-net [29] is a frequently used fully convolutional network with skip connections between the downsampling and upsampling paths. These methods have successfully been used for ventricle segmentation [22, 30] and WMH segmentation [23–26] separately. They generate results in a fraction of the time of the conventional methods mentioned above [17], which is important when analysing big data sets and for use in clinical settings, and they can easily incorporate multi-contrast information for greater accuracy [26, 30]. However, it would be beneficial if methods performed well using a variety of imaging contrasts in case of missing MRI sequences. CNNs are often trained on large sets of labeled training data and may not produce as accurate results when applied to images in data sets with different scanning protocols, MRI scanners, or subject populations [31]. A manual delineation by an expert in neuroanatomy is still the gold standard for ground truth segmentations. Obtaining manually segmented images is laborious and slow, and hence often impractical for generating new training data for different data sets.

We recently developed the Segmentation AutoEncoder (SegAE [32]), an unsupervised CNN method for segmentation of the grey matter (GM), white matter (WM), cerebrospinal fluid (CSF), and WMHs in brain MRIs without the need for manually annotated training data. We have previously shown that this method produces robust and accurate WMH segmentations on multi-site data [32]. However, the parcellation of the ventricular system into its four main compartments, i.e., the left and right lateral ventricles, and the 3rd and 4th ventricles, depends on human-made naming conventions so manually created atlases are needed to label these structures.

Here we propose a novel segmentation pipeline comprising a sequential use of SegAE and a ventricle parcellation CNN, which in conjunction with ventricle labels automatically generated by a multi-atlas segmentation approach, provides a joint WMH and ventricle segmentation. First, SegAE is used to generate images that represent the proportion of GM, WM, CSF, and WMHs in each voxel. These are combined into a standardized image with relatively homogeneous intensities within each tissue class. A ventricle parcellation network, hereafter referred to as the Ventricle CNN or V_CNN, is trained using the standardized images as input for robustness to changes in protocol between data sets. We train the SegAE network on T1-w, T2-w, and FLAIR images to generate the standardized images without using any training labels. The V_CNN is then trained on standardized images and corresponding ventricle labels generated by the multi-atlas segmentation approach RUDOLPH [14]. Two major advantages are gained from using the standardized image instead of the raw MRI sequences as input: First, with the standardized image, we have a single image with sharp tissue contrast enabling us to fit larger patches into GPU memory than if multiple images were used as input (i.e., using the chosen patch-size, a larger number of input channels would not fit into GPU memory); and second, SegAE can produce images with standardized contrast using MRIs from different scanners or from only a selection of available sequences (e.g., T1-w only or T1-w and T2-w only), which can be beneficial in data sets where some of the sequences are not available (see Section Input sequence dependence for details).

The segmentation of WMHs and the parcellation of the ventricular system into the four compartments is validated using ground truth manually delineated labels from two different data sets: The Age, Gene/Environment Susceptibility (AGES) Reykjavik study, a unique longitudinal study of the Icelandic elderly [33]; and an NPH cohort from the Johns Hopkins Hospital, with mild to severe ventriculomegaly, to test the robustness of the method to multi-site data and severe pathology. We then apply the proposed method to process a total of 2401 subjects, aged 66–93 years old, from the AGES-Reykjavik cohort, including 90 subjects from the development set. We explore the ventricle volume and WMH load compared to age and sex to demonstrate the importance of taking both the ventricles and WMHs into account when analysing brains of elderly subjects and show how information from a large population of normal subjects can be used to identify abnormal ventricle enlargement or WMH load. For this analysis we use two separate multiple linear regression models to explore the association between ventricle volume and WMH load as well as age, sex, body mass index (BMI), CSF (excluding ventricles), intracranial volume (ICV), blood pressure, hypertension medication, diabetes mellitus, and smoking. This may provide clues as to which populations are most at risk of WMHs or ventricle enlargement and when a robust segmentation of both WMHs and the ventricles is essential.

## Materials

### AGES-Reykjavik study

The AGES-Reykjavik study was initiated in 2002 and was designed to examine risk factors, including genetic susceptibility and gene/environment interaction, in relation to disease and disability in old age [33]. The AGES-Reykjavik study cohort comprises 5764 participants (female and male, age 66–93 at first visit), 4811 of which underwent brain MRI [34]. A total of 2644 out of the 4811 subjects had a second visit on average 5 years later. The MRIs were acquired using a dedicated General Electrics 1.5-Tesla Signa Twinspeed EXCITE system with a multi-channel phased array head cap coil. T1-w three-dimensional (3D) spoiled gradient echo sequence (time to echo (TE): 8 ms, time repetition (TR): 21 ms, flip angle (FA): 30°, field of view (FOV): 240 mm; 256 × 256 matrix) with 0.94 × 0.94 × 1.5 mm^3^ voxel size and 110 slices; Proton Density (PD)/T2-w fast spin echo sequence (TE1: 22 ms, TE2: 90 ms, TR: 3220 ms, echo train length: 8, FA: 90°, FOV: 220 mm^2^; 256 × 256 matrix); and FLAIR sequence (TE: 100 ms, TR: 8000ms, time from inversion (TI): 2000 ms, FA: 90°, FOV: 220 mm; 256 × 256 matrix) with 0.86 × 0.86 × 3.0 mm^3^ voxel size and 54 slices.

#### Development set

For developmental purposes we selected 90 subjects (age 67–92) from the AGES cohort. These subjects were selected based on previously reported total ventricle volumes [34]. The quality of this ventricle segmentation was not assessed systematically, however, it was sufficient to roughly group subjects into three groups of 30: Group 1 containing the smallest, Group 2 the medium, and Group 3 the largest ventricle sizes. This way our development sample covered the entire spectrum of ventricle sizes of the AGES cohort (smallest to largest). Out of the development set of 90 subjects, 60 subjects were used for training, 5 for validation of model parameters, and the remaining 25 were used for testing. Each of these subsets were randomly selected, stratified by each ventricle group.

### NPH patients

A second data set from the Johns Hopkins Hospital, Baltimore, USA was used to test the robustness of the proposed method to a different scanner type and subject population. Brain MRIs of 80 NPH patients (age range 26–90 years with average age 66.8±15) were acquired with a 3-Tesla scanner. MPRAGE sequence (TR: 2110 ms, TE: 3.24 ms, FA: 8°, TI: 1100 ms) with a 0.9 mm isotropic voxel size, axial T2-w sequence (TR: 6500 ms, TE: 134 ms, TA: 2:38) with a 3 mm slice thickness, and an axial FLAIR sequence (TR: 9000, TE: 94 ms, TI: 2500 ms, TA: 2:44) with a 3 mm slice thickness.

#### Development set

The selection of the SegAE training subjects from the NPH data set was performed by rating the severity of WMHs in the 80 NPH subjects on a scale from 0 to 3 and randomly selecting 10 images with a severity of 3. To determine the hyperparameters for SegAE, 3 subjects were randomly selected for validation. The remaining 77 subjects (including the 10 training subjects) were used for testing the quality of the ventricle segmentation. Out of the subjects with a WMH score of more than 0, 10 subjects were randomly selected for testing the quality of the WMH segmentation.

### Preprocessing

The images in the AGES-Reykjavik data set and the NPH data set were pre-processed by resampling to 0.8 × 0.8 × 0.8 mm^3^ voxel size using cubic spline interpolation, rigidly registering the baseline T1-w images to the MNI-152 atlas space [35] and, in turn, registering the baseline T2-w, FLAIR and follow-up images to the corresponding baseline T1-w images in the MNI-152 atlas space. All images were skullstripped using the skullstripping U-net described in S1 File. Since inhomogeneity correction is a part of the training process for SegAE (as described in [32]), no inhomogeneity correction was needed during pre-processing.

## Methods

**Fig 1:**
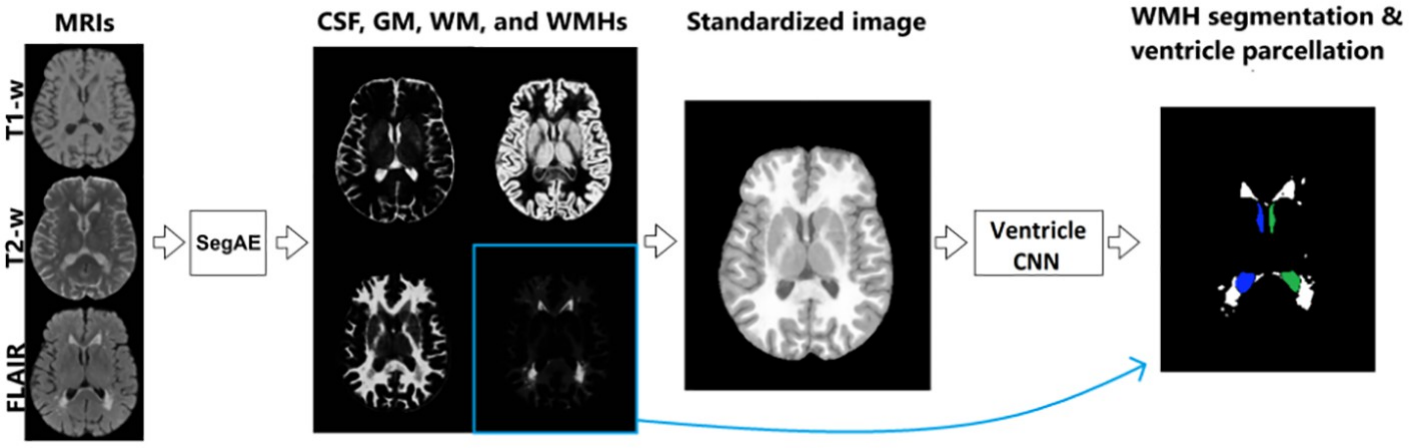
The proposed pipeline for joint ventricle and WMH segmentation. SegAE is used to decompose T1-w, T2-w and FLAIR images into four images, where the proportion of CSF, GM, WM, and WMHs is represented in each voxel. These are in turn used to create a standardized image from which the Ventricle CNN parcellates the ventricular system into the left and right lateral ventricles, and the 3rd and 4th ventricles.

Two different CNN architectures were used for two sequential tasks in the pipeline; the segmentation autoencoder (SegAE) for unsupervised tissue and WMH segmentation and a U-net specifically designed for parcellating the ventricular system (i.e., the V_CNN) using standardized images made from the SegAE segmentations as input. Fig 1 shows the complete segmentation pipeline. Pretrained weights for the pipeline, and code, programmed in Python and with the Keras/Tensorflow framework, are publicly available on GitHub. (See https://github.com/lmellingsen/Ventricle_WMH_segmentation/).

### Ethics statement

The data underlying the results of this study come from a large ongoing retrospective study of medical records and archived samples in Iceland (AGES-Reykjavik study). Participants underwent a broad written informed consent, approved by the Icelandic National Bioethics Committee and the Icelandic Data Projection Authority, that allows future studies to be performed on this data set without additional Institutional Review Board applications if they fulfil certain restrictions. Our study was specifically approved by the Data Regulatory Board of the Icelandic Heart Association to meet those criteria. All data were fully anonymized before we were given access to this widely used database. All protocols for the NPH data were approved by the Institutional Review Boards at the Johns Hopkins University School of Medicine. For all study participants, written informed consent was obtained and all data were fully anonymized before we were given access.

### Generation of training data and CNN architecture

SegAE, described in [32], is a CNN architecture that learns tissue and lesion segmentation in an unsupervised manner, by reconstructing multi-contrast MRI sequences as weighted combinations of the predicted tissue proportions. Using appropriate regularization and iterative inhomogeneity correction during training, the tissue proportions converge to a meaningful classification that represents WMHs and tissue classes. We then generate segmentations of the WMHs, GM, WM, CSF, and the meninges (the meninges were discarded in subsequent steps), using the T1-w, T2-w, and FLAIR images as input to SegAE.

**Fig 2:**
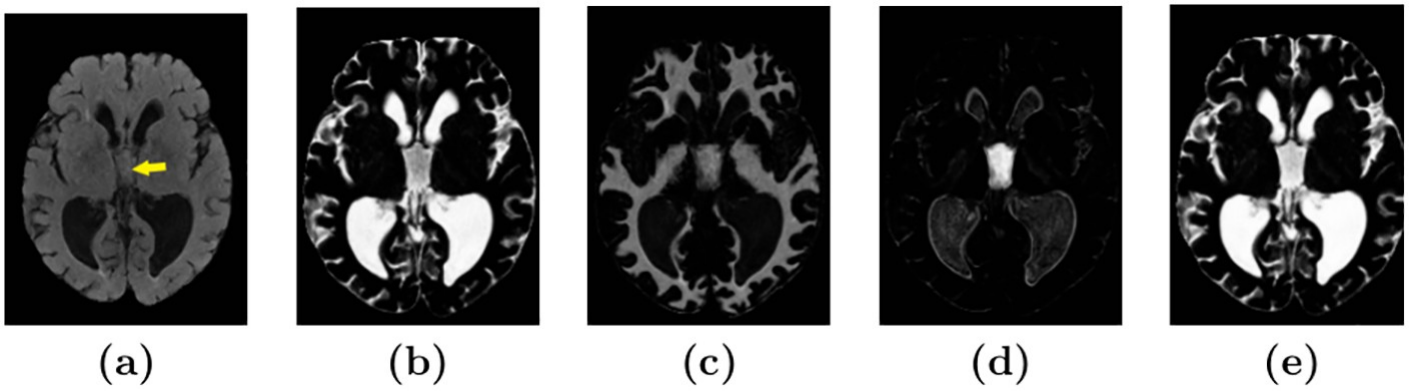
Identification and removal of pulsation artifact. Image **(a)** shows a FLAIR image with a pulsation artifact in the third ventricle (yellow arrow). Images **(b)** and **(c)** show the corresponding CSF and WMH output, respectively, from SegAE before thresholding. Image **(d)** shows a pulsation artifact segmentation obtained with element-wise multiplication of the CSF and WMH outputs (non-binarized). Image **(e)** shows the CSF segmentation that has been corrected for pulsation artifacts by adding the pulsation artifact segmentation shown in **(d)**.

The resulting CSF segmentations sometimes contained unwanted signal decay due to pulsation artifacts, which appeared bright as WMHs within the ventricles in FLAIR images (see the 3rd ventricle in Fig 2(a)). This sometimes resulted in the pulsation artifact being classified as WMHs. We corrected for these artifacts using a pulsation artifact segmentation obtained with an element-wise multiplication of the CSF and WMH segmentations from SegAE. The pulsation artifact segmentation was then added to the CSF segmentation and subtracted from the WMH segmentation. Results from this correction procedure are shown in Fig 2.

**Fig 3:**
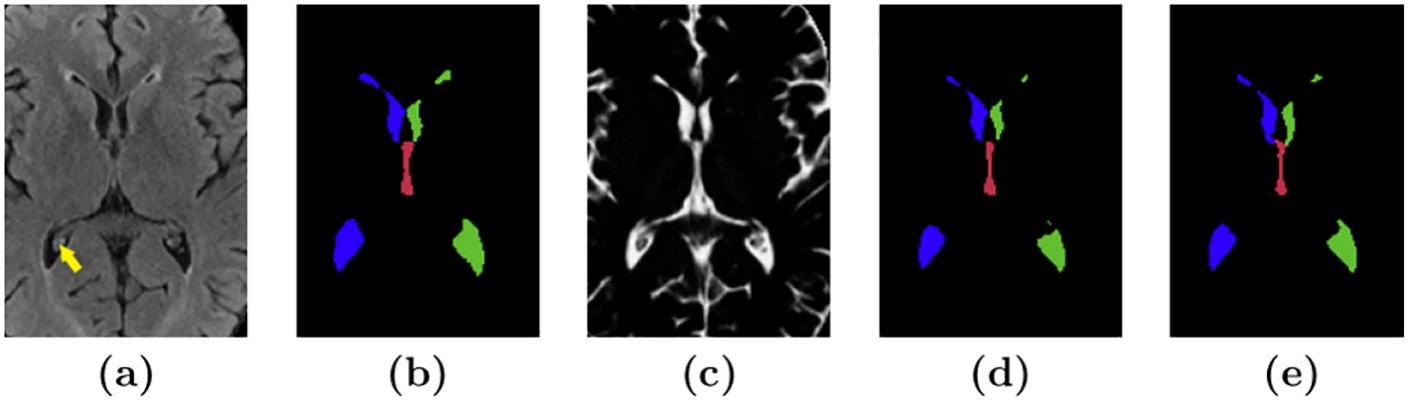
Preparation of training labels. Image **(a)** shows a slice of a FLAIR image showing choroid plexus in the right lateral ventricle (yellow arrow). Image **(b)** shows the corresponding ventricle segmentation from RUDOLPH, and **(c)** shows the corresponding CSF segmentation from SegAE. Image **(d)** shows a ventricle segmentation obtained by element-wise multiplication of each label from RUDOLPH with the CSF segmentation in **(c)** and a morphological closing of the lateral and third ventricles. **(e)** shows a corresponding manual delineation.

RUDOLPH [14] is a hybrid multi-atlas segmentation method that was specifically developed for subjects with hydrocephalus. The method combines multi-atlas segmentation and patch-based tissue classification in a special relaxation scheme that corrects the anatomical atlas priors in regions where accurate registration of the images is unachievable due to severely enlarged ventricles. RUDOLPH provides a robust segmentation and parcellation of the ventricular system into the left and right lateral, third, and fourth ventricles. We ran the RUDOLPH algorithm on the development set of 90 subjects from the AGES-Reykjavik data set and the ventricle labels for the left and right lateral ventricles and the 3rd and 4th ventricles were isolated. The RUDOLPH ventricle labels corresponding to subjects in the training set were manually inspected and in 25 images, lateral ventricle labels erroneously appearing in the sulcal CSF were manually removed. Subsequently, the CSF segmentation from SegAE was multiplied with the RUDOLPH ventricle labels to generate parcellated ventricle training labels that were consistent with the tissue segmentation from SegAE. This improved the quality of the training labels due to RUDOLPH’s consistent over-segmentation of the ventricles. The new ventricle labels were further processed with morphological closing to fill holes in the segmentation of the ventricles due to the brighter choroid plexus within the ventricles (see Fig 3). Morphological closing was performed with a 3 × 3 × 3 cube for two iterations in the lateral and third ventricles.

**Fig 4:**
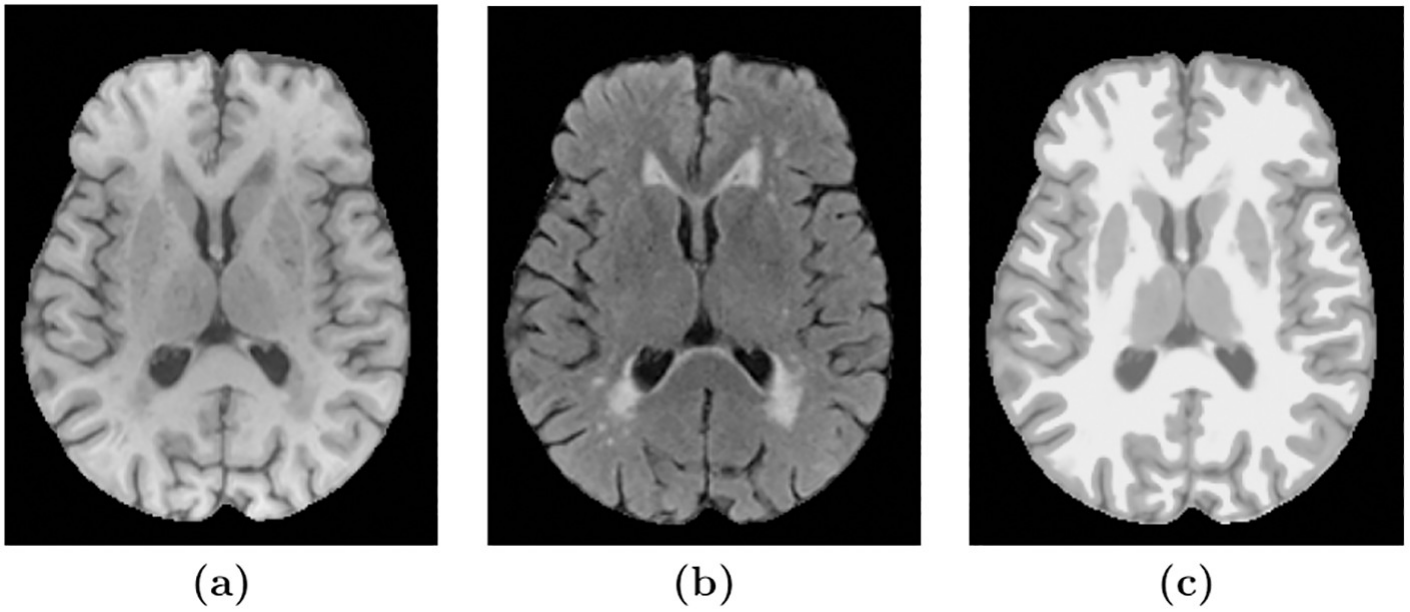
The MRI sequences vs. the standardized image. Images **(a)** and **(b)** show T1-w and a FLAIR images of a subject, respectively, and image **(c)** shows a standardized image made of SegAE segmentations, which is free of inhomogeneity artifacts and WMHs.

**Fig 5:**
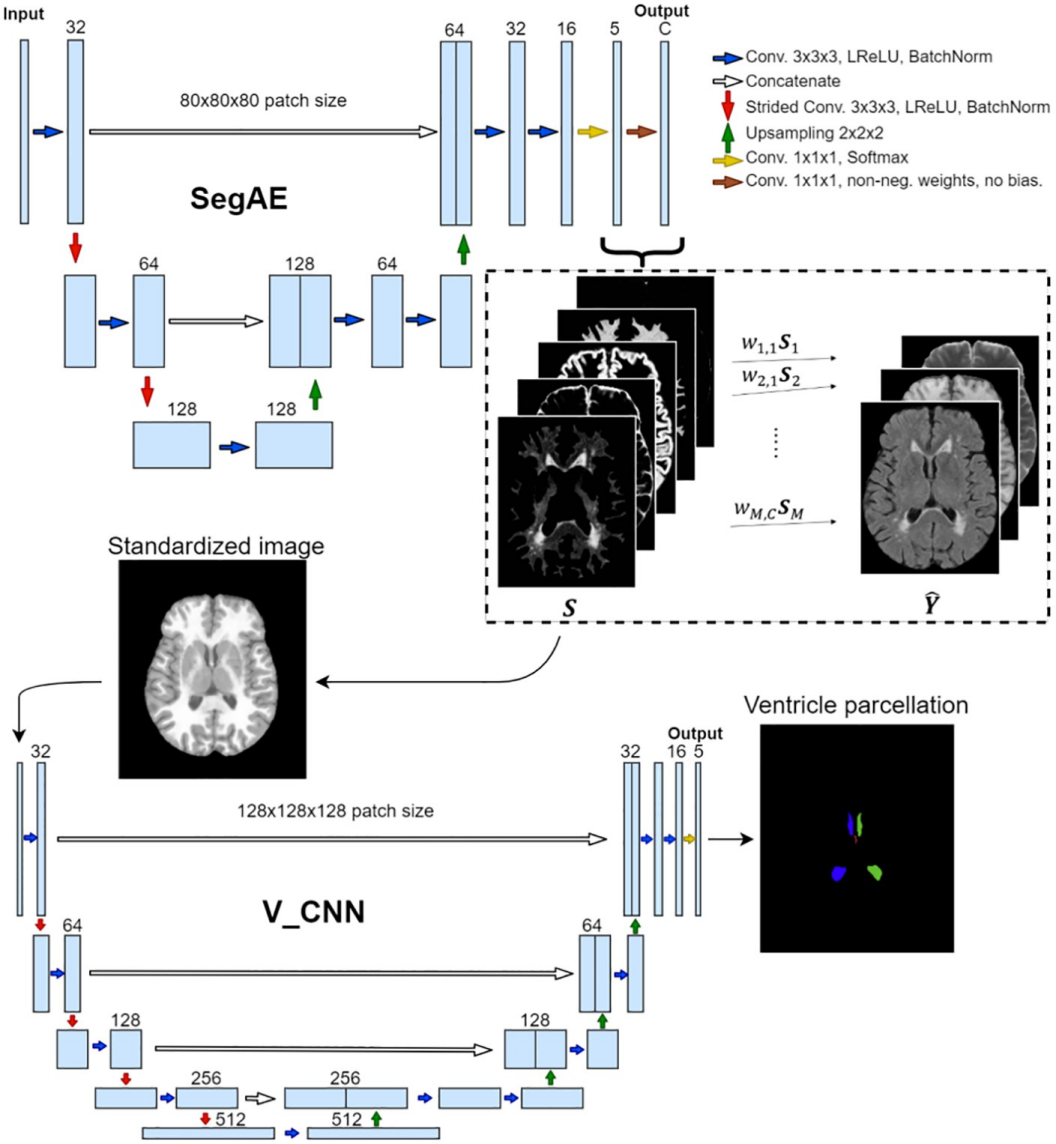
The proposed CNN architecture. The input into the SegAE network comprises large 3D patches of MRI images that are in turn reconstructed in an unsupervised manner by SegAE (the reconstructed output is denoted with $\hat{Y}$). The estimated components of the reconstruction (denoted with S) provide the segmentation of the input into WMHs, WM, GM, CSF, and meninges (meninges are discarded in subsequent steps), which in turn are used to create a standardized image that is used as the input into the V_CNN. Kernels of size 3 × 3 × 3 are used in all convolutional layers except size 1 × 1 × 1 is used in the final two layers of both SegAE and the V_CNN. The V_CNN output is a segmentation of the four ventricle compartments, which in conjunction with the SegAE output provides a consistent ventricle and WMH segmentation.

The input into the ventricle segmentation network was a weighted combination of the soft segmentation outputs from SegAE

$$\begin{array}{c} I_{standard}=1\cdot S_{CSF}+2\cdot S_{GM}+3\cdot \left(S_{WMH}+S_{WM}\right) \end{array}$$

where *I*_*standard*_ is the standardized image and *S*_*CSF*_, *S*_*GM*_, *S*_*WMH*_ and *S*_*WM*_ are the soft segmentations of the CSF, GM, WMHs, and WM, respectively. Scalar multiplication (⋅) with the weights 1, 2, and 3 is used to distinguish the tissues when they are combined into one image. Doing this allows us to use a single homogeneous image with a sharp tissue contrast as input (see Fig 4(c)), fit larger patches into GPU memory than if all the SegAE segmentations or MRI sequences were used as input, and to standardize tissue contrast when different sequences or MRIs from different scanners are used. The CNN architecture for the combined SegAE and V_CNN pipeline can be seen in Fig 5.

### Training and prediction

#### AGES-Reykjavik data set

The SegAE network was trained using T1-w, T2-w and FLAIR images from 30 subjects, as described in [32]. For the evaluation of input sequence dependence in Section Input sequence dependence, two other trained SegAE networks were prepared using the same method: One SegAE network was trained using only T1-w images as input, and another using only T1-w and T2-w images as input to the network. All three SegAE networks still use the same three inhomogeneity corrected MRI sequences, i.e. T1-w, T2-w and FLAIR, for calculation of the loss function during initial training, since inhomogeneity correction is a part of the training process for SegAE (as described in [32]). However, after the SegAE network has been trained, the missing input sequences for the two special networks are not needed for prediction of new subjects (see details of this sequence dependence experiment in Section Input sequence dependence).

Standardized images were created from the SegAE segmentations of the AGES-Reykjavik training set and 128 × 128 × 128 voxel patches were extracted with a 40 voxel stride. The V_CNN was trained on standardized images and corresponding ventricle labels from 60 subjects. The SegAE network trained using T1-w and T2-w images as input, did not show the strong pulsation artifacts that were apparent in the SegAE CSF segmentation when FLAIR images were used as input. Therefore, the SegAE network using only T1-w and T2-w images as input was used for post-processing of the RUDOLPH ventricle labels that were used for training the V_CNN. The V_CNN was trained using a Dice loss for 200 epochs (due to memory constraints, 15 subjects were selected 4 times to train for 50 epochs) with a learning rate of 1⋅10^6^, using the Adam optimizer [36] with Nesterov momentum [37] with *β*_1_ = 0.9, *β*_2_ = 0.999, schedule decay of 0.004, and a batch size of one. The learning rates were chosen manually with six tries and comparisons with the validation set of 5 subjects (labels generated in the same way as the training data in Section Generation of training data and CNN architecture). Other hyperparameters were not changed from the default values of Tensorflow [38]. After training, ventricle label prediction was performed with a stride of 64, and patches were assembled using the average of overlapping voxels. The training scheme is summarized in Table 1.

**Table 1:** The training scheme for the two CNNs of the pipeline, SegAE and V_CNN, for the two data sets, i.e., the AGES-Reykjavik cohort and the NPH cohort.

	AGES-Reykjavik input	NPH input
SegAE	Unsupervised training using T1-w, T2-w/PD-w, and FLAIR images	Unsupervised fine-tuning using T1-w, T2-w, and FLAIR images
V_CNN	Supervised training using automatically generated labels from RUDOLPH and standardized images from SegAE	No training– network trained using standardized images derived from the AGES-Reykjavik data

#### NPH data set

The SegAE network trained on the AGES-Reykjavik images was further trained using T1-w, T2-w and FLAIR images of the 10 training subjects in the NPH data set using a learning rate of 0.0001. The V_CNN that was trained on the AGES-Reykjavik data set was used directly on the NPH data set, with no retraining, and prediction was performed in the same way as for the AGES-Reykjavik data. An automatic post-processing of the ventricle parcellation was performed by changing sporatic lateral- and 3rd ventricle labels in the same connected component as the fourth ventricle.

## Results

We present three experiments for the proposed joint ventricle and WMH segmentation method. First, we conduct a comparison with widely used, publicly available segmentation methods, using manual delineations as a reference. Second, we experiment with different combinations of input sequences into our segmentation pipeline. Finally, we compare the segmented volumes to various biomarkers in the AGES-Reykjavik data set and explore the strength of association between ventricle size and WMH load.

### Evaluation and comparison

The four ventricle compartments and WMHs in a total of 25 subjects (8–9 from each Group of different ventricle sizes described in Section AGES-Reykjavik Study) from the AGES-Reykjavik cohort were manually delineated for evaluation of the proposed method. In addition, the method was evaluated on 77 subjects with manual ventricle labels and 10 subjects with manual WMH labels from the NPH data set. For both of these data sets, the entire ventricular system was labeled first as a single binary mask from the T1-w image. Then each ventricle mask was parcellated into the left and right lateral ventricles, and the 3rd and 4th ventricles. The WMHs were manually segmented from the FLAIR images.

All test subjects were processed using the proposed method, as well as two whole brain segmentation methods: The widely used FreeSurfer 6.0 [13] and RUDOLPH, which was specifically developed to be robust to severely enlarged ventricles. Furthermore, the method was compared with two publicly available and widely used WMH segmentation methods: LGA [39] and LPA [40].

FreeSurfer is a comprehensive software package for analysis of structural and functional neuroimaging data, including labelling of cortical and sub-cortical brain structures and WMHs. The WMH segmentation method LGA segments WMHs from T1-w and FLAIR images. A CSF, GM and WM segmentation is first obtained from the T1-w image and combined with FLAIR image intensities for calculation of WMH belief maps, which are subsequently thresholded (*κ* = 0.1 and 0.5, for the AGES-Reykjavik data and NPH data respectively, determined using the validation sets) and grown to include hyperintense FLAIR voxels for a final lesion probability map. The WMH segmentation method LPA segments WMHs from FLAIR images, using a logistic regression model, trained on MRIs of 53 MS patients with severe lesion patterns obtained at the Department of Neurology, Technische Universität München, Munich, Germany. A lesion belief map and a spatial covariate, accounting for voxel specific changes in lesion probability, are used. Finally, the lesion probability map can be thresholded for a WMH segmentation. These four segmentation methods could be applied to our data sets without the need for a new set of manually delineated training segmentations. We ran FreeSurfer both with default parameters and with the -bigventricles flag to account for enlarged ventricles.

For each subject in the test set the following metrics were computed to evaluate the performance of the proposed method and alternative methods compared to the manually delineated structures:

- *Dice Similarity Coefficient (DSC)*
  A measure of overlap between the ground truth and predicted segmentations, DSC is defined as $2\frac{\left|A\cap B\right|}{\left|A|+|B\right|}$, where **A** and **B** are binary masks. A DSC of 1 indicates a perfect overlap and 0 indicates no overlap between **A** and **B**.
- *Log Volume Ratio (LVR)*
  A log transformed ratio of the predicted volume *V*_*P*_ to the true volume *V*_*T*_. LVR is defined as $log(\frac{V_{P}}{V_{T}})$. Lower LVR indicates a more accurate prediction.
- *Lesion-wise F1-score (L-F1)*
  Let *N*_*P*_ be the number of correctly detected lesions after comparing the predicted lesion mask *P* to the ground truth lesion mask *T*. *N*_*F*_ is the number of incorrectly detected lesions in *P*. An individual lesion is defined as a 3D connected component, and L-F1 is defined as $\frac{N_{P}}{N_{P}+N_{F}}$. Higher L-F1 indicates better performance.
- *Modified Hausdorff distance (H95)*
  Hausdorff distance measures the longest distance one has to travel from a point in one set to a point in the other set, defined as:
  $$\begin{array}{c} d_{H}\left(X,Y\right)=\max\left\{\;\underset{x\in X}{\text{sup}}\underset{y\in Y}{\text{inf}}\;d\left(x,y\right),\;\underset{y\in Y}{\text{sup}}\underset{x\in X}{\text{inf}}\;d\left(x,y\right)\;\right\}\text{,}\; \end{array}$$
  where *d*(*x*, *y*) denotes the distance between *x* and *y*, sup denotes the supremum and inf the infimum. Here the 95th percentile is used instead of the maximum distance, since the Hausdorff distance is sensitive to outliers. Lower H95 scores indicate better performance.

**Fig 6:**
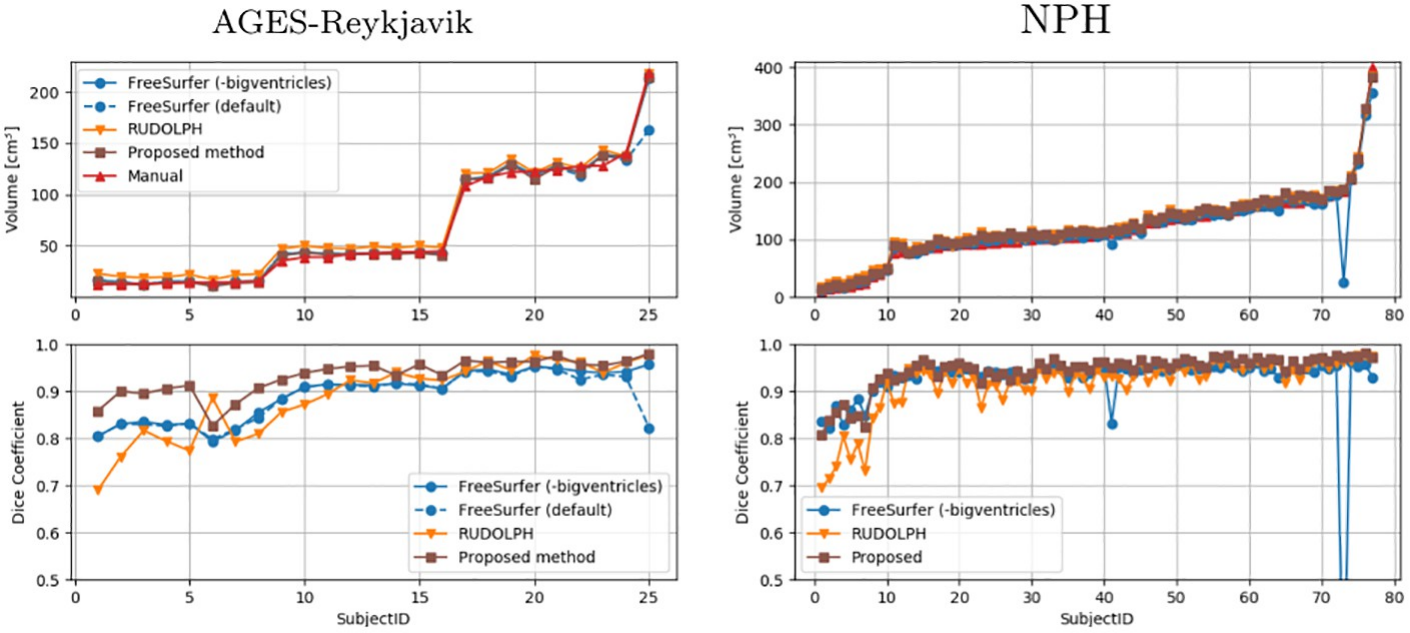
Quantitative evaluation of the ventricle segmentation. The top graphs show the overall ventricle volume for the manual masks (red) and masks generated by FreeSurfer (blue), RUDOLPH (orange), and the proposed method (brown), ordered by the volume of the manual masks. The bottom graphs show the DSC for the same methods compared with the manual masks. Results on the AGES-Reykjavik data are shown on the left and the NPH data on the right.

Fig 6 shows the ventricle volumes (of the entire ventricular system combined) of the manual masks and the estimated ventricle volumes using the three methods, as well as the DSC between the corresponding ventricle segmentations and the manual masks, ordered by the volume of the manual masks. This way, the performance of the methods relative to ventricle volume is demonstrated, since the DSC is known to be sensitive to the size of the segmented volume [41]. The proposed method shows the most stable performance on all three groups of ventricle sizes in the AGES-Reykjavik data set, and achieves the highest DSC on 20 out of the 25 subjects. RUDOLPH has the lowest DSC score on the smallest ventricles. FreeSurfer with default settings fails when presented with the largest ventricles. In subsequent analysis we omit results from default FreeSurfer, since FreeSurfer with the -bigventricles flag has a similar performance as the default version on subjects with smaller ventricles. The proposed method shows a stable performance on all 77 subjects with NPH compared to FreeSurfer, which shows poor performance on two subjects with severely enlarged ventricles, and RUDOLPH, which has a consistently lower DSC score.

Table 2 shows the DSC, LVR, and H95 metrics for the entire ventricular system and each sub-compartment. Table 3 shows the DSC, LVR and L-F1 metrics for the WMHs. Scores are averaged over all subjects and the best scores are shown in bold. Statistical significance was determined using a Wilcoxon signed-rank test and values that are significantly different from the proposed method are denoted with an asterisk (*). The proposed method achieves the highest average DSC and H95 scores on the entire ventricular system (significantly better than FreeSurfer and RUDOLPH on both the AGES-Reykjavik and NPH data sets). On the NPH data set, the proposed method also achieves the highest LVR score (significantly better than FreeSurfer and RUDOLPH), however, FreeSurfer achieves a slightly better LVR score on average than the proposed method on the AGES-Reykjavik data set, although it is not significantly different. The corresponding scores for the left- and right lateral ventricles and the 3rd and 4th ventricles seperately are also shown in Table 2. The proposed method achieves the highest average WMHs segmentation score on all three metrics compared to FreeSurfer, LGA, and LPA on the AGES-Reykjavik data set. The L-F1 score is sensitive to lesions with high-overlap splitting into parts, or a few voxels connecting otherwise unconnected lesions in the ground truth segmentation. LPA achieves the highest DSC and L-F1 scores on the NPH data sets, although, was not significantly better than the proposed method. Our method achieved the best LVR score, which was not significantly different from the comparison methods.

**Table 2:** Evaluation of the ventricle segmentation. The mean and standard deviation of the DSC, LVR, and H95 for FreeSurfer, RUDOLPH and the proposed CNN pipeline on the entire ventricular system (Entire), the left lateral ventricle (LLV), the right lateral ventricle (RLV), the third ventricle (3rd) and the fourth ventricle (4th). A paired Wilcoxon signed-rank test was used to obtain the p-values for determining statistical significance.

		AGES-Reykjavik data set (N = 25)		
		FreeSurfer	RUDOLPH	Proposed
Entire	DSC	0.894 (± 0.048)*	0.888 (± 0.079)*	**0.932 (± 0.038)**
	LVR	**0.071 (± 0.072)**	0.200 (± 0.171)*	0.072 (± 0.068)
	H95	6.939 (± 7.229)*	6.624 (± 8.384)*	**2.816 (± 5.408)**
LLV	DSC	0.906 (± 0.044)*	0.889 (± 0.081)*	**0.938 (± 0.039)**
	LVR	0.072 (± 0.077)	0.203 (± 0.175)*	**0.070 (± 0.077)**
	H95	7.155 (± 8.370)*	7.451 (± 0.175)*	**2.942 (± 5.459)**
RLV	DSC	0.900 (± 0.052)*	0.890 (± 0.081)*	**0.935 (± 0.038)**
	LVR	**0.073 (± 0.070)**	0.195 (± 0.179)*	0.078 (± 0.074)
	H95	7.646 (± 0.916)*	7.205 (± 9.222)*	**3.848 (± 7.750)**
3rd	DSC	0.853 (± 0.044)	0.867 (± 0.056)	**0.869 (± 0.039)**
	LVR	**0.136 (± 0.098)**	0.188 (± 0.132)*	0.152 (± 0.119)
	H95	**2.260 (± 0.781)**	2.540 (± 0.993)	2.573 (± 0.910)
4th	DSC	0.687 (± 0.077)*	0.777 (± 0.092)*	**0.824 (± 0.054)**
	LVR	0.525 (± 0.191)*	0.417 (± 0.224)*	**0.199 (± 0.144)**
	H95	12.857 (± 3.231)*	2.901 (± 1.434)	**2.615 (± 1.473)**
NPH data set (N = 77)				
Entire	DSC	0.923 (± 0.088)*	0.916 (± 0.060)*	**0.944 (± 0.036)**
	LVR	0.076 (± 0.227)*	0.110 (± 0.130)*	**0.074 (± 0.064)**
	H95	3.884 (± 5.788)*	17.564 (± 7.175)*	**2.562 (± 2.303)**
LLV	DSC	0.928 (± 0.084)*	0.921 (± 0.062)*	**0.945 (± 0.036)**
	LVR	0.073 (± 0.214)*	0.104 (± 0.134)	0.083 (± 0.068)
	H95	3.540 (± 5.493)*	17.820 (± 7.419)*	**2.180 (± 1.709)**
RLV	DSC	0.925 (± 0.097)*	0.915 (± 0.059)*	**0.946 (± 0.034)**
	LVR	0.079 (± 0.276)*	0.108 (± 0.126)*	**0.073 (± 0.038)**
	H95	3.883 (± 6.404)*	20.394 (± 7.936)*	3.999 (± 7.977)
3rd	DSC	0.830 (± 0.076)	**0.851 (± 0.095)**	0.837 (± 0.104)
	LVR	0.155 (± 0.169)*	0.234 (± 0.215)	**0.219 (± 0.238)**
	H95	3.512 (± 1.852)	2.642 (± 1.321)*	4.192 (± 5.308)
4th	DSC	0.739 (± 0.078)*	**0.805 (± 0.070)**	0.775 (± 0.127)
	LVR	0.417 (± 0.179)*	**0.309 (± 0.189)**	0.315 (± 0.364)
	H95	9.771 (± 3.815)	2.885 (± 1.493)*	8.583 (± 7.499)

Asterisk (*) denotes values that are significantly different from the proposed CNN (*p* < 0.05/15, where 15 corrects for multiple comparisons), and bold figures denote the best score for each metric.

**Table 3:** Evaluation of the WMH segmentation. The mean and standard deviation for DSC, LVR, and L-F1 for the WMH segmentations from FreeSurfer, LGA, LPA, and SegAE. A paired Wilcoxon signed-rank test was used to obtain the p-values for determining statistical significance.

	AGES-Reykjavik data set (N = 25)			
	FreeSurfer	LGA	LPA	Proposed
DSC	0.284 (± 0.107)*	0.634 (± 0.146)*	0.669 (± 0.175)	**0.774 (± 0.100)**
LVR	0.697 (± 0.255)*	0.322 (± 0.352)	0.558 (± 0.607)*	**0.297 (± 0.307)**
L-F1	0.127 (± 0.068)*	0.309 (± 0.117)*	0.354 (± 0.185)	**0.437 (± 0.085)**
	NPH data set (N = 10)			
	FreeSurfer	LGA	LPA	Proposed
DSC	0.482 (± 0.0.120)*	0.665 (± 0.110)	**0.778 (± 0.053)**	0.721 (± 0.070)
LVR	0.754 (± 0.288)	0.634 (± 0.233)	0.360 (± 0.156)	**0.334 (± 0.173)**
L-F1	0.088 (± 0.037)	0.086 (± 0.020)	**0.163 (± 0.057)**	0.146 (± 0.070)

Asterisk (*) denotes values that are significantly different from the proposed CNN (*p* < 0.05/12, where 12 corrects for multiple comparisons), and bold figures denote the best score for each metric.

**Fig 7:**
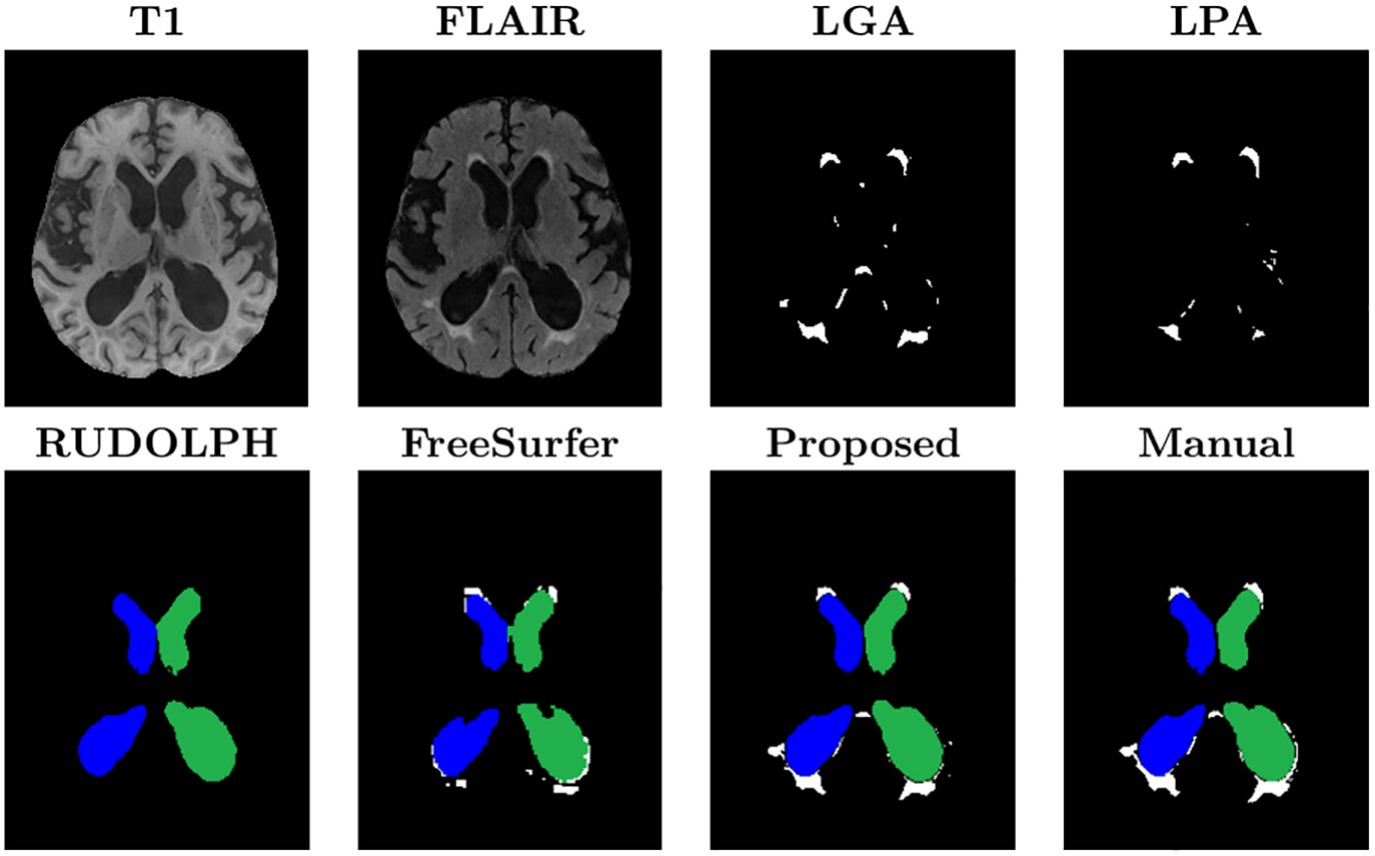
Visual comparison of the proposed method and the five methods used for comparison. The images show the left (green) and right (blue) lateral ventricles (the 3rd and 4th ventricles are not visible in these slices), and WMHs (white). LPA and LGA provide WMH labels but not ventricle labels. RUDOLPH and FreeSurfer provide a whole brain segmentation with ventricle labels, however RUDOLPH does not provide WMH labels and the WMH labels from FreeSurfer are not accurate. The proposed method provides accurate ventricle and WMH labels.

A visual comparison of the ventricle and WMH segmentations from the proposed method and the alternative segmentation methods can be seen in Fig 7. LPA and LGA provide WMH labels but not ventricle labels. RUDOLPH and FreeSurfer provide a whole brain segmentation with ventricle parcellation, however RUDOLPH does not provide WMH labels, as is common in multi-atlas segmentation approaches, and the WMH labels from FreeSurfer are not accurate, as expected, given that FreeSurfer’s segmentation is entirely based on the T1-w sequence, where WMHs have similar intensity values to GM structures. The proposed method is able to provide accurate and consistent (i.e., non-overlapping labels) WMH and ventricle segmentation, while FreeSurfer’s WMH labels tend to bleed into the labels of the lateral ventricles.

### Input sequence dependence

**Fig 8:**
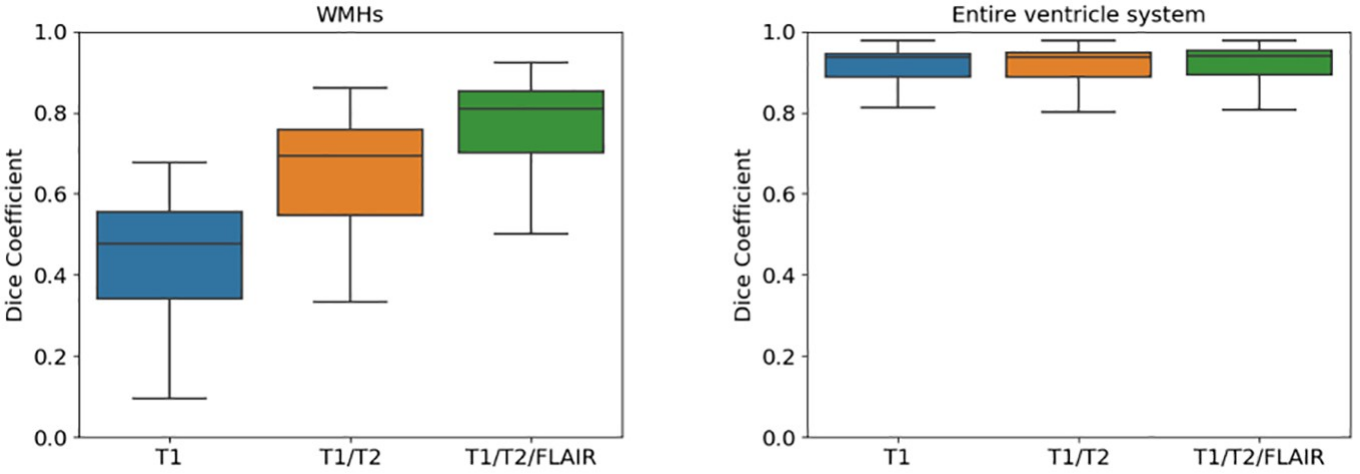
Boxplots comparing the DSC when using different number of input sequences in the proposed method. The left plot shows WMHs and the right plot shows the ventricular system when generating segmentations from: 1) Only T1-w (blue), 2) only T1-w and T2-w (orange), and 3) T1-w, T2-w, and FLAIR images (green).

In our second experiment we wanted to explore whether the proposed method was able to segment the WMHs and the ventricles using fewer input sequences than the network was trained on. This would be beneficial, for example, for WMH segmentation when FLAIR images are missing. Three SegAE networks trained on the AGES-Reykjavik data set were used for this experiment, one using only the T1-w image as input, another using T1-w and T2-w images as input, and one using T1-w and T2-w and FLAIR images as input. Only one V_CNN network was used to segment the ventricles in standardized images created with segmentations from the three SegAE networks separately. The AGES-Reykjavik test set was used for evaluation of the input sequence dependence. Fig 8 shows boxplots of the DSC coefficients for WMHs (Fig 8, left) and the entire ventricular system (Fig 8, right) for the proposed method when using the following sequences as input: 1) only T1-w images, 2) only T1-w and T2-w images, and 3) using T1-w, T2-w, and FLAIR images as input. As expected, the segmentation accuracy for WMHs is not as accurate when some of the sequences are missing, however, we note that our method is able to produce similar WMH segmentation results as the LGA method but without using the FLAIR image as input (mean DSC 0.647±0.140 for the proposed method vs. 0.634±0.146 for LPA). Likewise, the proposed method produced a better average DSC for the WMH segmentation than FreeSurfer when using only the T1-w image as input (mean DSC 0.442±0.162 for the proposed method vs. 0.284±0.107 for FreeSurfer). For the ventricle segmentation, there is no significant difference in accuracy when using one, two, or all three sequences.

### Association between ventricle size and lesion load

In our final experiment, we explored associations between the segmentation volumes and various biomarkers in the AGES-Reykjavik study on data from 2371 subjects. We ran the pipeline on 2401 subjects for which all the required data existed and omitted 30 subjects due to failures in processing (24 due to registration errors and 6 due to skullstripping errors).

**Fig 9:**
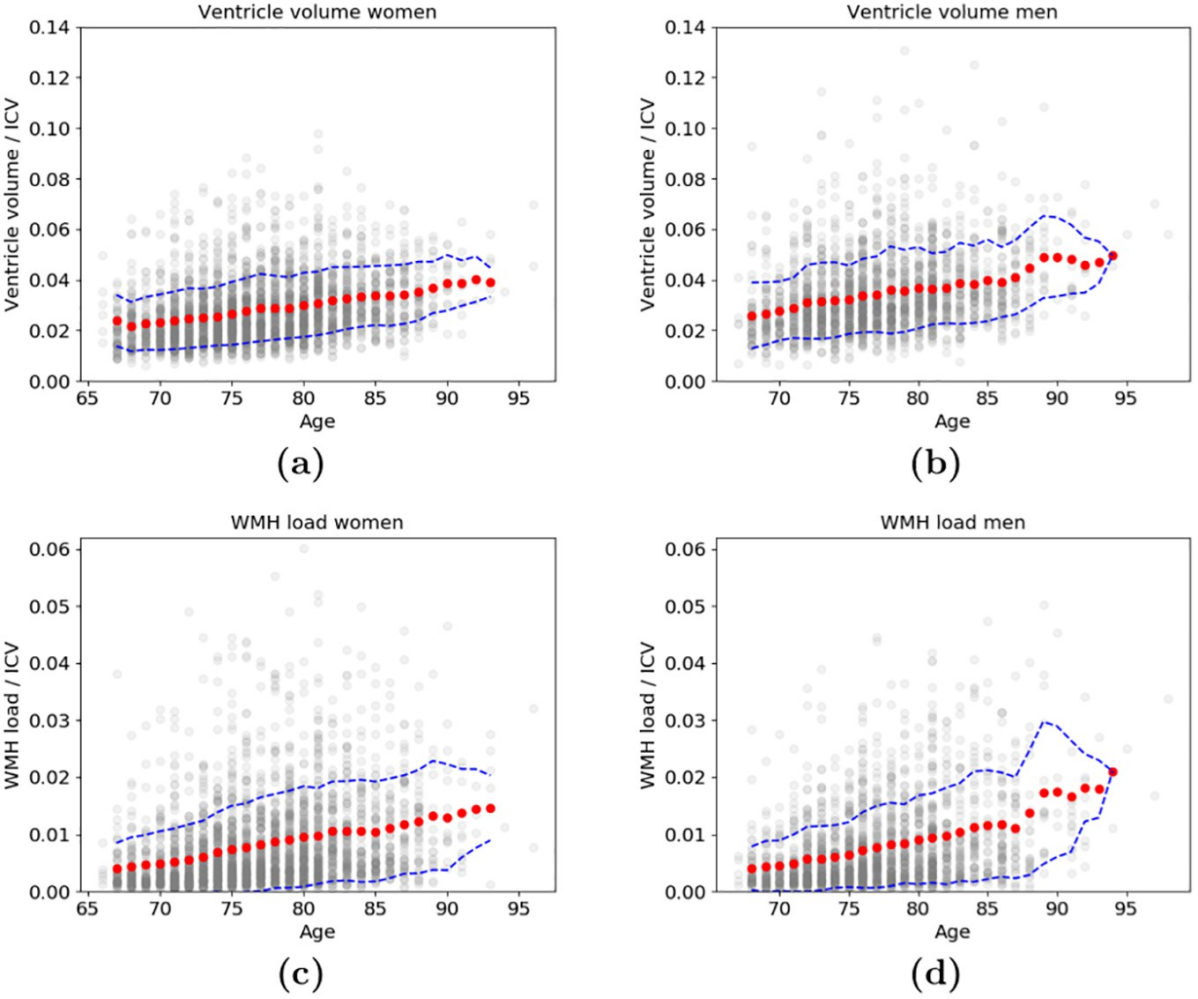
The 3 year moving average (red dots) and standard deviation (dashed blue line) of ventricle volumes and WMH load. The association between age and the total volume of all the ventricles divided by ICV (top) for **(a)** women and **(b)** men, as well as the association between age and WMH load divided by ICV (bottom) for **(c)** women and **(d)** men. The ventricle volume and WMH load of individual subjects, at their corresponding age, are shown in grey.

First, we explored the relationship of age and the total volume of the ventricles divided by ICV as well as the WMH load divided by ICV for men and women (see Fig 9). We show the 3 moving year average and standard deviation of the volumes for each age group. While there was an overall increase in both ventricle volume and WMH load with age, the individual variability was high within each age group.

Second, we compared selected segmentation volumes from our pipeline (ventricle- and sulcal CSF volumes, WMH load, and ICV) to several demographics and biomarkers in the AGES-Reykjavik study (see Table 4) to explore risk factors for either ventricle enlargement or high WMH load and the individual association between the two. These biomarkers were carefully selected as they have previously been shown to be associated with WMHs and enlarged ventricles. Table 5 shows the results from two multiple linear regression models on data from 2371 subjects at first visit (baseline) to predict the volume of the entire ventricular system (Table 5, top) and the WMH load (Table 5, bottom), respectively. The adjusted coefficients of determination (adjusted-*R*^2^) of the two models were 0.141 and 0.275, for the WMH prediction and the ventricle volume prediction, respectively. The ventricle model has the WMH load as input, and the WMH load model has the entire ventricle volume as input. Furthermore, both models include the sulcal CSF volume, ICV, age, sex, body mass index (BMI), systolic and diastolic blood pressure, use of hypertension medication, diabetes mellitus type 2, and history of smoking. The values were normalized by subtracting the mean and dividing by the standard deviation. The use of hypertension medication and the presence of diabetes mellitus type 2 is represented with the dichotomous variables 0 and 1, for absence and presence, respectively. Smoking status is represented with the categorical variables 0, 1, and 2 for non-, former-, and current smoker, respectively. Using the multiple regression model we found that WMHs, ICV, age, and diabetes mellitus type 2 are significantly associated with the ventricle volume (*p* < 0.05/22, where 22 corrects for multiple comparisons). Furthermore, we found that ventricle volume, age, sex, diastolic blood pressure and smoking are significantly associated with WMH load (*p* < 0.05/22, where 22 corrects for multiple comparisons). Thus, there may be other underlying reasons for the association between WMHs and ventricle volume than increasing age and the other biomarkers and demographic factors mentioned above.

**Table 4:** Demographics and biomarkers [mean and standard deviation (SD)] at baseline for the 2371 subjects used in the multiple regression model.

	Baseline
**Age [mean, SD]**	74.7, 4.8
**Sex [% male]**	41%
**Body mass index (BMI) [mean, SD]**	27.2, 4.1
**Systolic blood pressure [mean, SD]**	141.3, 19.9
**Diastolic blood pressure [mean, SD]**	74.1, 9.3
**Hypertension medication [count]**	1430
**Diabetes mellitus type 2 (DM2) [count]**	214
**History of Smoking [count]**	[0: 1026, 1: 1091, 2: 254]

**Table 5:** Multiple linear regression models to predict the entire ventricle volume (top) and WMH load (bottom). Input parameters are given on the left, followed by the regression coefficients (*β*_*n*_), the standard error (*S*), the t statistic and p-values, as well as the 95% confidence interval.

	**Predicting ventricle volume**					
	*β*_*n*_	*S*	*t*	*p*	[0.025	0.975]
**Constant**	40.7473	0.355	114.688	0.000	40.051	41.444
**WMH load**	4.1334	0.375	11.028	0.000	3.398	4.868
**sulcal CSF volume**	0.5497	0.477	1.152	0.250	-0.386	1.486
**Intracranial volume (ICV)**	6.9992	0.536	13.052	0.000	5.948	8.051
**Age**	3.3780	0.413	8.177	0.000	2.568	4.188
**Sex**	-1.3190	0.506	-2.609	0.009	-2.310	-0.328
**Body mass index (BMI)**	0.6169	0.367	1.681	0.093	-0.103	1.337
**Systolic blood pressure**	0.3602	0.423	0.851	0.395	-0.470	1.190
**Diastolic blood pressure**	-0.0727	0.429	-0.170	0.865	-0.913	0.768
**Hypertension medication**	-0.0216	0.369	-0.059	0.953	-0.746	0.703
**Diabetes mellitus type 2**	1.1835	0.365	3.246	0.001	0.469	1.898
**History of smoking**	0.3159	0.368	0.859	0.391	-0.406	1.037
	**Predicting WMH load**					
	*β*_*n*_	*S*	*t*	*p*	[0.025	0.975]
**Constant**	9.3225	0.180	51.742	0.000	8.969	9.676
**Ventricle volume**	2.2806	0.207	11.028	0.000	1.875	2.686
**sulcal CSF volume**	0.1301	0.242	0.537	0.591	-0.345	0.605
**Intracranial volume (ICV)**	0.5573	0.281	1.981	0.048	0.006	1.109
**Age**	1.8497	0.209	8.850	0.000	1.440	2.260
**Sex**	0.9884	0.256	3.862	0.000	0.487	1.490
**Body mass index (BMI)**	0.2958	0.186	1.589	0.112	-0.069	0.661
**Systolic blood pressure**	0.4248	0.215	1.980	0.048	0.004	0.845
**Diastolic blood pressure**	0.7570	0.217	3.491	0.000	0.332	1.182
**Hypertension medication**	0.5315	0.187	2.843	0.005	0.165	0.898
**Diabetes mellitus type 2**	0.0216	0.185	0.117	0.907	-0.342	0.385
**History of smoking**	0.9274	0.186	4.996	0.000	0.563	1.291

## Discussion

Our hybrid multi-atlas segmentation and convolutional autoencoder approach jointly provides a segmentation of WMHs and a parcellation of the ventricular system into its four main compartments. First, a segmentation of the WMHs, CSF, WM, and GM is acquired with the unsupervised CNN method SegAE. Then the training labels for the ventricle parcellation network, i.e., the V_CNN, are acquired without manual delineation by merging labels from the multi-atlas segmentation method RUDOLPH with the CSF segmentation provided by SegAE. The input to the V_CNN is a standardized image created with a linear combination of the SegAE segmentations.

We compared with methods that are publicly available. Furthermore, given that our method requires no manually delineated ground truth data, we selected methods for comparison that do not require training on manually delineated ground truth data in a new data set. Our results imply that standardized images allow us to segment brain structures, such as the ventricles, using different types of sequences as input to the pipeline. In contrast, if a CNN was trained using the MRI sequences directly, different CNNs would have to be trained for each combination of input sequences [42] or by incorporating image synthesis [43]. Doing that would limit the method to data sets with similar MRI parameters and scanner characteristics. This method can serve as an alternative to training on ground truth from multiple data sets at once or as an alternative to domain adaptation techniques for translating images between different domains [44]

### Generalizability

Simply by training an unsupervised tissue and WMH segmentation method on the NPH data set, the V_CNN trained only on AGES-Reykjavik data could be used to further parcellate the ventricular system in standardized images of NPH subjects. This data set was especially challenging because of severely enlarged ventricles and, in many cases, strong pulsation artifacts in the FLAIR images. Our method is a step towards making CNNs, trained in a supervised manner using manually delineated labels or labels from multi-atlas segmentation methods, able to directly segment new brain MRI data (using different scanners or protocols) without the need to generate new training labels.

Pulsation artifacts were removed from the CSF and WMH segmentations from SegAE using a pulsation artifact mask obtained with element-wise multiplication of the soft CSF and WMH segmentation masks. A limitation of this approach is that if the pulsation artifact is too strong, such that it is exclusively present in the WMH segmentation, the multiplication will be zero. A pulsation artifact output could potentially be incorporated into SegAE to generalize the method further and avoid pulsation artifacts affecting the CSF and WMH segmentations.

**Fig 10:**
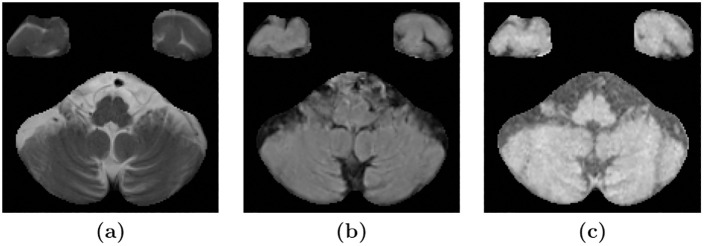
Resampling may cause erroneous WMH segmentation in the cerebellum. Images **(a)**, **(b)**, and **(c)** show an axial slice of the cerebellum in T2-w, FLAIR, and T1-w images, respectively, of a subject in the NPH data set. The T2-w images have a higher in-plane resolution, which shows the thin lines of CSF in the cerebellum. Meanwhile, the upsampling of the lower resolution FLAIR and T1-w images gives them a blurry appearance, leading to brighter voxels instead of fine dark lines corresponding to the CSF in the T2-w image.

We have demonstrated the generalizability of the method to different data sets by training the method on data from the AGES-Reykjavik cohort and then applied the method on the challenging NPH data set without any manually delineated training labels. A more extensive validation can be done with access to other manually delineated, multi-contrast data sets with ventricle and WMH lesion labels. One limitation that we came across when inspecting the SegAE WMH segmentations of the NPH data set was sporadic WMH labels erroneously appearing in the cerebellar region in some subjects of the NPH data set. We believe that this is due to resampling during pre-processing. Resampling may create a problem for unsupervised multi-contrast methods such as SegAE when there is a large difference in resolution between the available MRI sequences. For instance, when thin lines of CSF in high-resolution T2-w images of the NPH data set correspond to brighter voxels in FLAIR and T1-w images due to blurring (see Fig 10). One solution could be to use the lower FLAIR resolution as a reference for registration as proposed in [45]. However, the ground truth manual delineations that existed for our data set were only available in MNI space. Future solutions may involve more advanced super-resolution techniques.

### Input sequence dependence

We conducted an ablation experiment to test if our method could be used to generate accurate segmentations without using all three MRI sequences as input (i.e., the T1-w, T2-w, and FLAIR images). Our method was shown to give a robust ventricle segmentation when changing the input MRI sequences used to generate the standardized images with SegAE (see Fig 8). Furthermore, we showed that the method could be used to segment WMHs without using the FLAIR images as input, i.e., by using only T1-w or only T1-w and T2-w images, although with some resignation in DSC. However, the DSC for the WMH segmentations when only T1-w and T2-w images were used as input were still comparable to the LPA method. Similarly, using only T1-w images as input, the average DSC was higher when using the proposed method rather than FreeSurfer. Therefore, this strategy may be a viable option in data sets where FLAIR images are not available for all subjects.

### Investigating associations

Finally, we conducted an experiment where we compared the ventricle and WMH segmentation volumes to various demographics and clinical biomarkers in the AGES-Reykjavik data set. The aim was to determine the variability in the elderly population and to explore the strength of association between ventricle sizes and WMHs and risk factors for both.

First we demonstrated in Fig 9 how the average ventricle sizes increase with age for both sexes and how a population data set could be used to determine enlarged ventricles using the standard deviation for each age group. Similarly, the average WMH load increases with age, and notably, the standard deviation also generally increases with age. Fewer data points between ages 90–97 cause the standard deviation to decrease. The results demonstrate the high variability of ventricle volumes and WMHs in the elderly population.

Ventricle volume and WMH load depend on multiple factors and to explore the individual association between the ventricle size and WMH load, we used multiple linear regression models that take several confounders into account. Previous studies have found hypertension to be a major risk factor for severe WMHs and that hyperintensive drugs reduce the risk of severe WMHs [46]. Our results showed a positive association with systolic and diastolic blood pressure, although only statistically significant for diastolic blood pressure, and a non-significant positive association with the use of hyperintensive drugs. The blood pressure variables were measured at the time of study and lack information about duration of high blood pressure over a longer time period for each subject. The use of hypertensive medication may indicate a longer history of high blood pressure and be positively associated with high WMH load even if they have a lowering effect on blood pressure. Diabetes mellitus type 2 has been associated with ventricle enlargement [47], as supported by our results, and a moderately elevated risk for lacunar infarction in older men [48]. Our results found a significant positive association with ventricle volume, but not with WMH load. Smoking has been associated with a higher WMH load [49], as seen in our results, however, we do not have an accurate measurement of how much or for how long each subject has smoked. Other lifestyle factors that are associated with smoking, such as alcohol consumption and/or less physical activity [50], may influence our results.

Previous studies have found associations between WMH load and region specific atrophy [51], which are both biomarkers of small vessel disease [52]. Previous analysis of the AGES-Reykjavik cohort have found that WMH and CSF volumes increase with age while the GM and WM volumes decrease [53], and disproportionate ventricular dilation is associated with WMH load [54]. The association of WMH load and ventricular volume has also been shown to be independent of demographics, vascular burden and APOE genotype [55]. In our results, the ventricle volume but not the sulcal CSF volume is associated with WMH load. There could be different causes for this in different individuals. WMHs possibly indicate reduced white matter integrity around the ventricles in some individuals, and perhaps the expansion of the ventricles might cause periventricular WMHs in the case of NPH patients [56]. Our results indicate that it is important to investigate the ventricles in the elderly, diabetes patients, and in people with small vessel disease; and WMHs in elderly people with high blood pressure, enlarged ventricles, and smokers.

The significant positive association between ventricle volume and WMH load indicate that there are other underlying reasons for this association (such as cerebral small vessel disease) than the variables used in the multiple linear regression models in Table 5. Simultaneous segmentation of both the ventricles and WMHs in large scale studies of the elderly population may shed further light on this connection and differentiate between causes of ventricle enlargement and increased WMH load [55], and how they contribute to dementia [57].

### Impact

The proposed method currently provides segmentation of WMHs and a detailed parcellation of the ventricular system, which has been a challenging task in the segmentation of brain MRIs of the elderly and people with neurodegenerative diseases [20, 58]. The method has the potential of being extended to include segmentations of other brain structures, both cortical and subcortical, that are usually segmented with methods that do not take WMHs or other tissue abnormalities into account (e.g., multi-atlas segmentation methods or supervised CNNs with labelled training data). The proposed method also enables us to segment directly from standardized images that can be created using different MRI protocols and scanners if appropriate measures are taken to correct for image artifacts.

## Conclusion

We have introduced a hybrid multi-atlas segmentation and convolutional autoencoder approach for a joint segmentation of WMHs and the four ventricular compartments in the human brain. The method was compared with the whole-brain segmentation methods FreeSurfer and RUDOLPH and the WMH segmentation methods LGA, and LPA. The proposed method achieved the best average DSC on the entire ventricular system in the AGES-Reykjavik cohort and in the NPH patient data set (comparison of individual ventricle structures and alternative metrics can be seen in Table 2). The proposed method achieved the highest average DSC, LVR and L-F1 for the WMH segmentation on the AGES-Reykjavik data set (see statistical significance in Table 3). LPA achieved the highest DSC and L-F1 for WMHs in the NPH data set (not significantly better than the proposed method), and the proposed method the best LVR (not significantly better than the comparison methods). We showed that WMH load and the ventricle volumes in the AGES-Reykjavik cohort are independently associated using a multiple linear regression model taking several potential confounders into account.

## Supporting information

S1 File A skullstripping U-net for brain MRIs. (PDF)
