# Supplementary material for: A joint ventricle and WMH segmentation from MRI for evaluation of healthy and pathological changes in the aging brain
Source: PLoS One. 2022 Sep 6;17(9):e0274212. doi: 10.1371/journal.pone.0274212 (PMC9447923; doi:10.1371/journal.pone.0274212)
Supplement: S1 File — (PDF) [file pone.0274212.s001.pdf]

A joint ventricle and WMH segmentation from MRI for evaluation of healthy and pathological changes in the aging brain: Supplementary material

Hans E. Atlason[a,*]

[a] *Dept. of Electrical and Computer Engineering, University of Iceland, Reykjavik, Iceland*

1. Skull-stripping U-net for brain MRIs

Isolating the intracranial matter from brain MRIs is important for subsequent processing in many image processing pipelines. Multiple methods have been developed for brain extraction [1, 2, 3], however their accuracy is often not consistent between subjects due to atrophy, enlarged ventricles, traumatic brain injury, or random errors. Atlas based methods are common, however, they often do not capture anatomical variability. One such brain extraction method is the Multi-contrast brain stripping (MONSTR) [4] method, which was developed to be robust to traumatic brain injury by utilizing multi-contrast information. Although highly accurate in most cases, we found that MONSTR fails in some subjects of the AGES-Reykjavik data set. State-of-the art methods for most brain segmentation tasks are based on convolutional neural networks (CNNs) [5, 6, 7]. They are usually more consistent because they are robust to random errors in the training set, which usually contains multiple manually delineated images. CNN based methods can also be much faster than methods based on multi-atlas registration with multiple degrees of freedom. However, manually delineated brainmasks are often not available and are time consuming to generate. Here we show that by training a three dimensional U-net CNN using the brainmasks generated by MONSTR as training data we can generate more accurate brainmasks than MONSTR, if images with the most visible errors are removed from the training set. This training method can be used when a skull-stripping method, such as MONSTR, generates near perfect brainmasks for a large

*See the main text for the complete author list

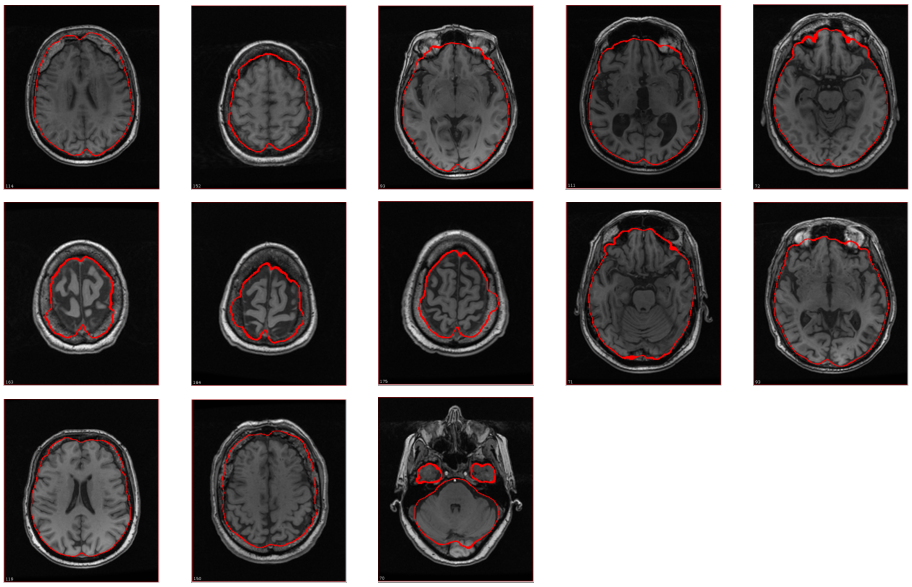

Figure 1: Erroneous skull-stripping results from MONSTR that were removed from our training set. The figure shows T1-w images and the corresponding skull-stripping boundaries generated by MONSTR (red).

subset of the data at hand, which can be used for training. Alternatively, brainmasks can be manually corrected, however, this is a much more time consuming approach.

1.1. Preparation of training data and CNN architecture

The development and evaluation of the skull-stripping U-net was performed using brain MRIs from the AGES-Reykjavik data set (cf. Section 2.1. in the main text). The brainmasks used for supervised training of the skullstripping CNN were generated by the MONSTR method [4]. Brainmask atlases for MONSTR were created by manually delineating the brain in 6 subjects from our AGES-Reykjavik development set of 120 subjects. Manual inspection of 60 of the generated MONSTR brainmasks led to the exclusion of 13 masks due to skullstripping failures (see Figure 1); hence the remaining 47 masks were used for training. Our training set comprised the T1-w, T2-w, and FLAIR images and the corresponding brainmasks. The network architecture can be seen in Figure 2.

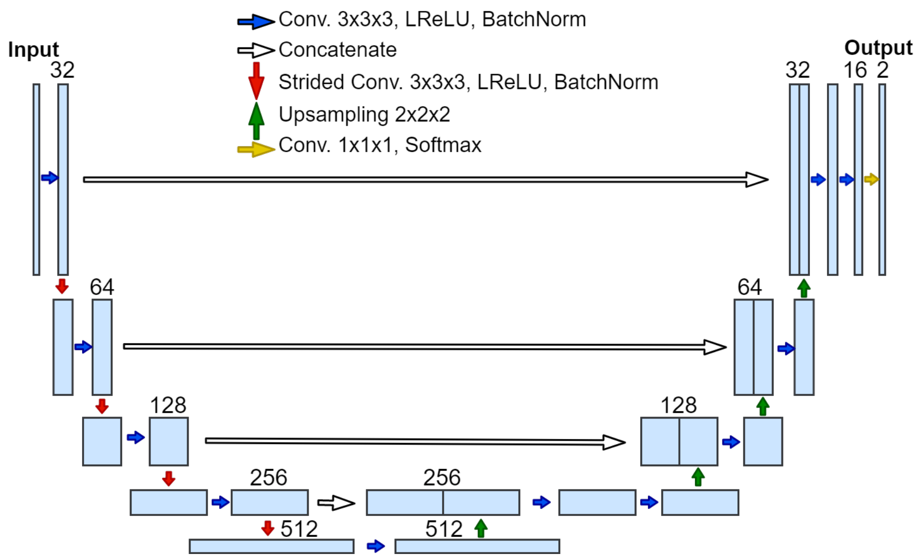

Figure 2: The proposed CNN architecture for the skullstripping U-net. The input comprises large 3D patches from FLAIR, T1-w, and T2-w images. Kernels of size 3×3×3 are used in all convolutional layers except size 1×1×1 is used in the final two layers.

1.2. Training

The 47 training images were intensity normalized by dividing by the 99th percentile of the non-zero elements of the image and 80×80×80 voxel patches were extracted with a 40 voxel stride. A weighted categorical cross-entropy loss function was used. The weights were determined with class weights [8]. The network was trained for 200 epochs with a learning rate of $1 \cdot 10^5$ using the Adam optimizer [9] with Nesterov momentum [10], with $\beta_1 = 0.9$, $\beta_2 = 0.999$, schedule decay of 0.004, and a batch size of 5.

1.3. Evaluation

The evaluation of our skull-stripping method was twofold: First, we compared the results of our method to results generated by MONSTR on the development set of 120 subjects. Second, we compared the intracranial volumes (ICVs) of 2401 subjects on MRI scans that were acquired at two different time points (scans acquired 5 years apart on average). We visually inspected 9 slices of each of these 2401 subjects to detect failures and their causes.

Figure 3 shows a histogram of the Dice dissimilarity (one minus the Dice similarity coefficient) between the 120 MONSTR brainmasks and the U-net brainmasks. Subjects with the lowest Dice dissimilarity between the

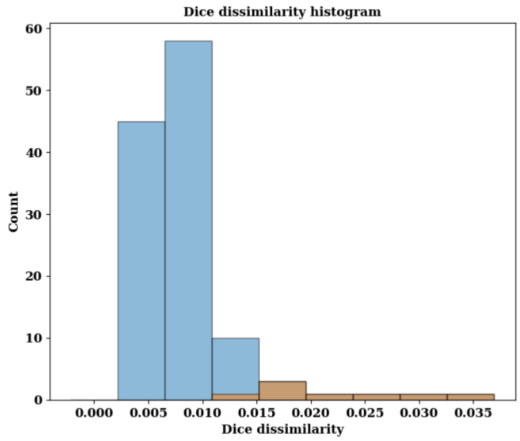

Figure 3: A histogram showing the number of the skullstripping U-net brain segmentations within each group of Dice dissimilarity when compared with the MONSTR segmentations. The brown columns show the values of the 8 brain segmentations with the highest dissimilarity and the rest is shown in blue.

U-net and MONSTR were selected one-by-one for visual comparison, until the visual differences between the two brain segmentations were negligible, resulting in 8 subjects. Figure 4 shows one slice from each of these 8 subjects that have the largest error.

One limitation to this evaluation strategy is that by comparing the overlap of the masks generated by the U-net to the masks from MONSTR we would not expect to find large values for Dice dissimilarity if both MONSTR and the U-net systematically fail in the same way. Therefore, we visually inspected 9 slices from each of the 2401 subjects with longitudinal MRI scans and found that the U-net was very robust, except in cases when: 1) One or more MRI sequences had registration errors (24 registration errors in total); and/or 2) there were visible skullstripping errors (9 cases of which 3 were caused by registration errors). These registration errors are marked on Figure 5, which compares the brainmask volumes at two timepoints. The figure shows that the predicted ICV is generally very consistent, with the exception of the few cases that had registration and/or skullstripping failures.

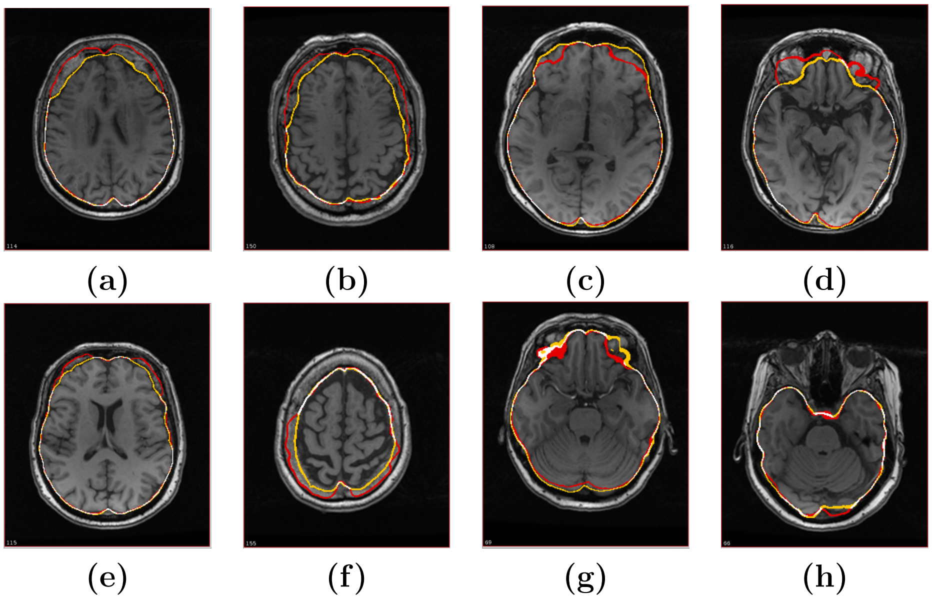

Figure 4: T1-w images and the corresponding skullstripping boundaries generated by MONSTR (red) and the U-net (yellow) and their overlap (white). Figures **(a)-(h)** show the 8 subjects from the development set that have the lowest Dice similarity in ascending order.

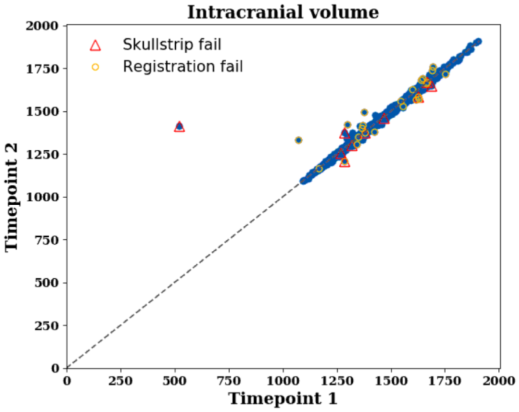

Figure 5: The intracranial volume predictions from the skullstripping U-net for MRIs at the first and second visit (timepoint 2 vs. timepoint 1) shown in blue. The line representing equal volume is shown with a dashed black line.

References

[1] M. Jenkinson, M. Pechaud, S. Smith, et al., Bet2: Mr-based estimation of brain, skull and scalp surfaces, in: Eleventh annual meeting of the

organization for human brain mapping, Vol. 17, Toronto., 2005, p. 167.

[2] S. F. Eskildsen, P. Coupé, V. Fonov, J. V. Manjón, K. K. Leung, N. Guizard, S. N. Wassef, L. R. Østergaard, D. L. Collins, A. D. N. Initiative, et al., Beast: brain extraction based on nonlocal segmentation technique, NeuroImage 59 (3) (2012) 2362–2373.

[3] J. E. Iglesias, C.-Y. Liu, P. M. Thompson, Z. Tu, Robust brain extraction across datasets and comparison with publicly available methods, IEEE transactions on medical imaging 30 (9) (2011) 1617–1634.

[4] S. Roy, J. A. Butman, D. L. Pham, Robust skull stripping using multiple MR image contrasts insensitive to pathology, Neuroimage 146 (2017) 132–147.

[5] C. Wachinger, M. Reuter, T. Klein, DeepNAT: Deep convolutional neural network for segmenting neuroanatomy, NeuroImage 170 (2018) 434–445.

[6] J. Kleesiek, G. Urban, A. Hubert, D. Schwarz, K. Maier-Hein, M. Bendszus, A. Biller, Deep MRI brain extraction: A 3d convolutional neural network for skull stripping, NeuroImage 129 (2016) 460–469.

[7] S. Roy, A. Knutsen, A. Korotcov, A. Bosomtwi, B. Dardzinski, J. A. Butman, D. L. Pham, A deep learning framework for brain extraction in humans and animals with traumatic brain injury, in: 2018 IEEE 15th International Symposium on Biomedical Imaging (ISBI 2018), IEEE, 2018, pp. 687–691.

[8] F. Pedregosa, G. Varoquaux, A. Gramfort, V. Michel, B. Thirion, O. Grisel, M. Blondel, P. Prettenhofer, R. Weiss, V. Dubourg, J. Vanderplas, A. Passos, D. Cournapeau, M. Brucher, M. Perrot, E. Duchesnay, Scikit-learn: Machine learning in Python, Journal of Machine Learning Research 12 (2011) 2825–2830.

[9] D. P. Kingma, J. Ba, Adam: A method for stochastic optimization, arXiv preprint arXiv:1412.6980 (2014).

[10] T. Dozat, Incorporating nesterov momentum into adam, `https://openreview.net/forum?id=OM0jvwB8jIp57ZJjtNEZ` (2016).
